# Cyclodextrin—Polymethylsilsesquioxane Combined System as a Perspective Iron Delivery System for Oral Administration

**DOI:** 10.3390/gels10090564

**Published:** 2024-08-30

**Authors:** Polina Orlova, Ivan Meshkov, Egor Latipov, Sergey Vasiliev, Ivan Mikheev, Daria-Maria Ratova, Alexandra Kalinina, Aziz Muzafarov, Irina Le-Deygen

**Affiliations:** 1 Department of Chemistry, Lomonosov Moscow State University, Moscow 119991, Russia; p.orlova2021@mail.ru (P.O.); mikheev.ivan@gmail.com (I.M.); darmarrat@gmail.com (D.-M.R.); 2 Enikolopov Institute of Synthetic Polymeric Materials, Russian Academy of Sciences (ISPM RAS), Moscow 117393, Russia; ivanbm@ispm.ru (I.M.); kalinina@ispm.ru (A.K.); aziz@ispm.ru (A.M.); 3 Institute of Nanotechnology of Microelectronics, Russian Academy of Sciences (INM RAS), Moscow 115487, Russia; la_019@mail.ru; 4 Federal Research Center of Problems of Chemical Physics and Medicinal Chemistry RAS (FRC PCP MC RAS), Chernogolovka 142432, Russia; viesssw@mail.ru

**Keywords:** anemia, cyclodextrin, hydrogels, drug delivery, polymethylsilsesquioxane

## Abstract

Anemia is a global health problem that affects both adults and children, but treatment is hampered by serious side effects, primarily associated with the gastrointestinal tract with oral administration of drugs. In this study, we aimed to develop an oral form of iron compounds using polymethylsilsesquioxane hydrogels. To boost loading efficiency and prolong release, the iron compounds (FeCl_3_ and ferrous D-Gluconate) are incorporated into a guest–host complex with 2-hydroxypropyl-beta-cyclodextrin. We used PRXD, SEM, EDX mapping, and FTIR to investigate the complex formation, as well as the incorporation of such complexes into hydrogels. The optimal system underlines a combination of ferrous D-Gluconate and HPCD in a 1:1 molar ratio, embedded into a hydrogel with a modest quantity of silicate crosslinks. We demonstrated the slowing of iron release in a gastric media. Mathematical investigation revealed that the Higuchi mechanism releases iron from the hydrogel.

## 1. Introduction

Today, diseases caused by iron deficiency, such as iron deficiency anemia (IDA), pose a global public health threat [1]. According to the World Health Organization, up to 42% of children under the age of 5 suffer from iron deficiency. In the structure of the incidence of IDA among adults, women of reproductive age play a significant role: according to WOS and UNICEF reports, up to 35% of women worldwide are affected by this disease. In clinical practice, intravenous drug administration is used for life-threatening IDA disorders [2], but in mass therapy, multiple oral formulations are used depending on the duration of treatment. On the market, iron component dosage formulations for oral administration range from iron salt drops (e.g., ferrous sulfate drops) to sucrosomal delivery systems (e.g., micronized iron pyrophosphate contained in a phospholipid shell [3]). However, the release of iron from these systems happens mostly in the stomach [2], resulting in significant side effects that decrease patients’ quality of life and contribute to inadequate adherence to therapy. In this situation, iron is absorbed into the body via the duodenal mucous membrane. Despite the fact that the drug’s residence time in the stomach and intestine differ significantly, the aggressive digestive environment in the stomach frequently causes premature release, making the task of designing a stimulus-sensitive delivery system for iron compounds for oral administration relevant.

Premature stomach release, along with reduced iron absorption, necessitates the development of innovative oral delivery mechanisms for iron complexes. Some research groups worldwide are working on this topic; one of the most current reviews [1] lists major promising iron delivery techniques, including liposomes, solid lipid nanoparticles [3,4], chelation [5], hydrogels, polymers, etc. A large number of studies are devoted to the inclusion of iron compounds in complexes with natural polysaccharides, for example, obtained from algae [6] and some plants, such as ginseng [7]. Several scientific groups have investigated mucoadhesive microspheres as a per oral drug delivery vehicle [8]. Such complexes are distinguished by high biocompatibility; however, since such systems are stabilized mainly by electrostatic interactions, accelerated release of iron compounds occurs in the stomach under acidic conditions.

Thus, delivery systems that are biocompatible yet provide controlled release of the drug are needed. There are special requirements for oral delivery systems: they must be inexpensive, biocompatible systems without a pronounced taste or smell. In the case of iron compounds, a separate requirement may be the prevention of staining of the teeth, which is especially true for oral therapy with salt solutions.

It is known that cyclodextrins are used to improve the biopharmaceutical properties of many drugs, including to mask an unpleasant taste and prevent undesirable effects in the oral cavity. There are literature data on the inclusion of iron ions in cyclodextrins. For example, Zheng and Tarr have synthetized Fe^2+^/carboxymethyl-β-cyclodextrin complexes and proved carboxyl groups of this cyclodextrin as binding sites of iron [9].

However, host–guest complexes without additional functionalization do not provide controlled drug release. The incorporation of such compounds into a gel matrix is a viable method for controlling the properties of the delivery system. Among all gels, attention is drawn to the class of Si-based gels [10], including silica gels and some other hydrogels, possessing high sorption capacity, biological inertness, biocompatibility, and low immunogenicity [11,12]. A significant advantage of such gels is their relatively simple synthesis (sol-gel method) [13]. The regulation of the synthesis conditions for such gels leads to the porous structure with desired characteristics: high adsorption capacity and a high degree of selectivity with respect to various substances, including biologically active molecules and pharmaceuticals.

One of the most promising approaches underlines applications of polymethylsilsesquioxane gels (PMSSO) [14], which are widely applied in medical practice as medical products. PMSSO-hydrogels do not interact with the blood coming through the gastrointestinal tract and are biocompatible and safe [15]. We have recently demonstrated that PMSSO hydrogels are indeed capable of sorbing iron salts such as FDA approved FeCl_3_ and Fe (II) D-gluconate [16]. However, there are two issues to be solved. (1) The efficiency of sorbing in the PMSSO hydrogel is not enough to produce an adequate therapeutic concentration and (2) the system PMSSO-ferrous D-gluconate in pilot experiments was prone to rapid drug release in acid media. 

In this work, we decided to overcome these issues by using the strategy of incorporating iron compounds into the tori of 2-hydroxypropyl-beta-cyclodextrin (HPCD). The resulting complexes are proposed to be incorporated into the PMSSO hydrogel network. So, here we are demonstrating the promising approach for an iron peroral drug delivery system: biocompatible PMSSO hydrogel is enriched by guest–host complexes of iron compounds and HPCD.

## 2. Results and Discussion

### 2.1. Structure and Physical Properties of Synthetized Hydrogels

PMSSO hydrogels are considered as a perspective drug delivery carrier for oral administration. Here we aimed to investigate the role of hydrogel structure in the sorption of ferrous-containing compounds. During the synthesis we have an opportunity to vary the density of crosslinking bonds inside the hydrogels and thus create more or less dense systems. We obtained 3 hydrogels: with dense crosslinks (links molar ratio 1:2, HG-2 sample), less dense crosslinks (links molar ratio 1:1, HG-1 sample) and with no extra inorganic crosslinks (silsesquioxane structure only, HG-3 sample). All sample names are presented in Table 1 for better representation.

PMSSO synthesis was conducted in accordance with the previously reported procedure [16]. Briefly, at the first step, alkaline hydrolysis of methyltriethoxysilane and heterofunctional condensation of intermediates with the formation of PMSSO sol was followed by partial neutralization of sol by acids with the formation of PMSSO hydrogel. ^29^Si NMR and IR spectroscopy confirmed the structure of synthetized hydrogels and have been discussed in detail in our previous work [16]. Examples of spectra are presented in the Supplementary section (Figures S1–S3). Synthetized hydrogels were characterized by means of classical protocols including the sorption capacity Congo red test and specific surface area analysis. Obtained values, presented in Table 2, indicate high sorption capacity for synthetized hydrogels and are in good agreement with previously published data [16]. By comparing synthetized hydrogels with other hydrogels obtained from synthetic [17] as well as natural [18] polymers, we confirm our hypothesis of high sorption capacity of PMSSO hydrogels. 

### 2.2. Physico-Chemical Studies of Complex Formation between Iron Salts and HPCD

Previously we have shown that PMSSO hydrogels are able to sorb iron salts, namely FeCl_3_ × 6H_2_O and ferrous D-gluconate [16]. The first one is a model system for the sorption of Fe^3+^ by hydrogels, while the second is a well-studied therapeutic applied in clinical practice. However, these systems are not stable. They demonstrate low sorption capacity corresponding to therapeutically important ferrous D-gluconate and can release the drug uncontrollably. On the other hand, the strategy of the triple system, hydrogel + HPCD + iron salt, is promising to achieve high sorption capacity as well as controlled release. 

Firstly, we had to study the interaction between HPCD and iron compounds. There are at least two main ways to obtain guest–host complexes with cyclodextrins: from the solutions and from the powders by means of kneading [19]. Kneading is a well-studied strategy to improve drug loading into guest–host complexes with cyclodextrins requiring high HPCD in excess for effective encapsulation [20], while mixture of solutions could be considered more convenient and rapid but it must be followed with freeze-drying. For FeCl_3_ × 6H_2_O, we have compared these ways for the molar ratio guest–host 1–5 to provide sufficient host excess. 

**Figure 1:**
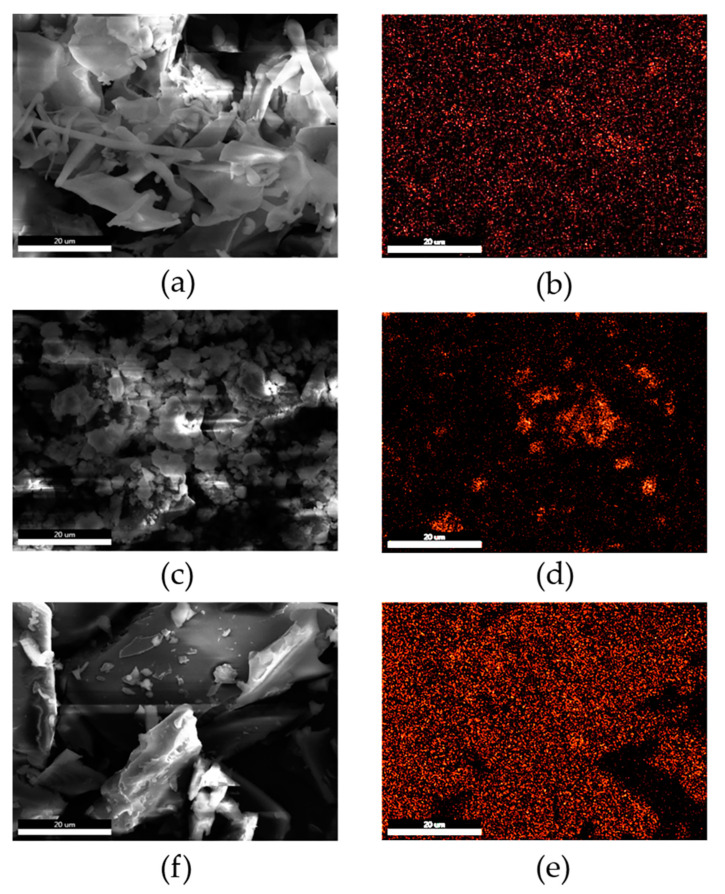
SEM (**a**) and EDX-map (**b**) of HPCD@FeCl_3_ sol; SEM (**c**) and EDX-map (**d**) of HPCD@FeCl_3_ kn; and SEM (**e**) and EDX-map (**f**) of HPCD@D-Glu sol.

Obtained from the solution complexes, HPCD@FeCl_3_ sol was studied in the solid phase after freeze-drying. Figure 1a,b displays SEM microphotographs and EDX mapping of the HPCD@FeCl_3_ sol inclusion complex. We have observed an even distribution of iron atoms in a medium amount in the sample, similar to the EDX mapping details for the PMSSO hydrogels loaded with FeCl_3_. PXRD data indicate the presence of an amorphous phase in both of the samples (Figure S4). On the other hand, samples from the kneading HPCD@FeCl_3_ kn demonstrates another pattern. According to the SEM microphotographs and EDX mapping (Figure 1c,d), the kneading technique leads to the higher efficiency of iron loading, but an uneven distribution of ions according to the EDX-mapping data. However, for the future therapeutics’ homogeneity and crucial reproducible loading, kneading does not seem to be a promising approach.

**Figure 2:**
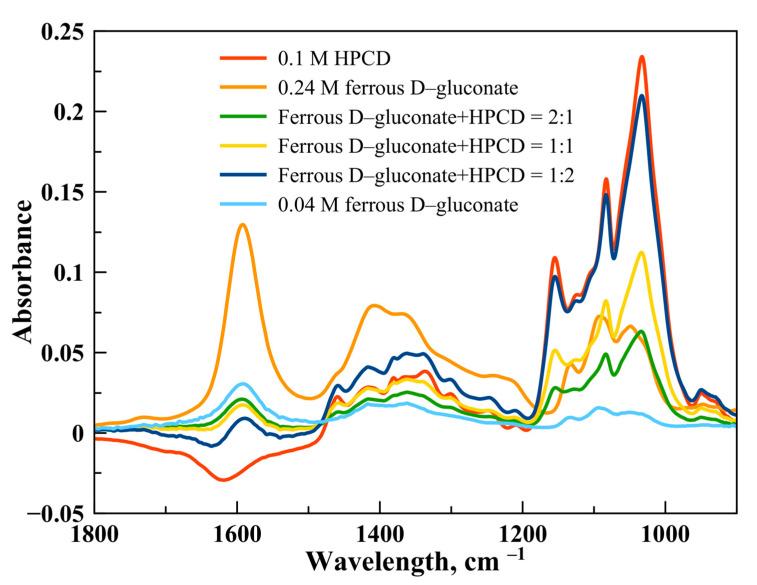
ATR-FTIR-spectra of 0.1 M HPCD (navy blue), 0.24 M ferrous D-gluconate (red), 0.04 M ferrous D-gluconate (yellow), and complex ferrous D-gluconate-HPCD in variable guest–host molar ratios: 2:1 (green), 1:1 (brown, named HPCD@D-Glu sol) and 1:2 (light blue), pH 4.0 (0.1 mM HCl), 22 °C.

On the other hand, the complex formation between HPCD and ferrous D-gluconate can be easily studied by means of ATR-FTIR spectroscopy in solutions due to the highly informative spectra of both compounds. Figure 2 represents the ATR-FTIR spectra of the solutions 0.1 M HPCD, 0.24 M and 0.04 M ferrous D-gluconate and complexes with varied guest–host molar ratios: 2:1, 1:1 and 1:2, typical for guest–host complexes with organic compounds [21]. 

For the ferrous D-gluconate spectrum, the following main bands were observed: 1593 cm^−^^1^ corresponding to ν COO^−^ vibration; 1360–1415 cm^−^^1^ corresponding to δ OH; and 1050–1130 cm^−^^1^ corresponding to ν C-O vibration. For the HPCD spectrum, we also observed 1360–1415 cm^−^^1^ corresponding to δ OH and 1050–1130 cm^−^^1^ corresponding to ν C-O vibration. As a result of complex formation, we also observed these bands, but the intensity of the 1593 cm^−^^1^ band (ν COO^−^) decreased after HPCD was added in comparison with the 0.04 M ferrous D-gluconate spectrum. At the same time, an increase in the content of HPCD causes a clear 1593 cm^−^^1^ intensity decrease and 1360–1415 cm^−^^1^ (δ OH) increase. From the obtained data follows that the intensity of 1050–1130 cm^−^^1^ (ν C-O) increases with the HPCD excess and also increases with the ferrous D-gluconate excess in the complex compared with the equimolar complex. Thus, complex formation occurs as a result of binding between COO^−^ and OH^−^; and OH^−^ from HPCD and OH^−^ from ferrous D-gluconate. 

The solution of the ferrous D-gluconate-HPCD 1:1 complex was freeze-dried and studied with PXRD and SEM methods (sample HPCD@D-Glu sol). As for previous samples, PXRD data indicate the presence of a mainly amorphous phase in the sample (Figure S5). Figure 1e,f demonstrates SEM microphotographs and EDX mapping of the sample HPCD@FeCl_3_ sol. We have observed an even distribution of iron atoms in the sample indirectly confirming complex formation. Moreover, the concentration of iron atoms is clearly higher than for HPCD@FeCl_3_ sol and kn samples. Thus, because of high loading capacity and more effective complex formation, for the further studies we have chosen ferrous D-Gluconate and HPCD@D-Glu. 

### 2.3. Study of the Ferrous D-Gluconate + HPCD Complexes’ Sorption by PMSSO Hydrogels

**Figure 3:**
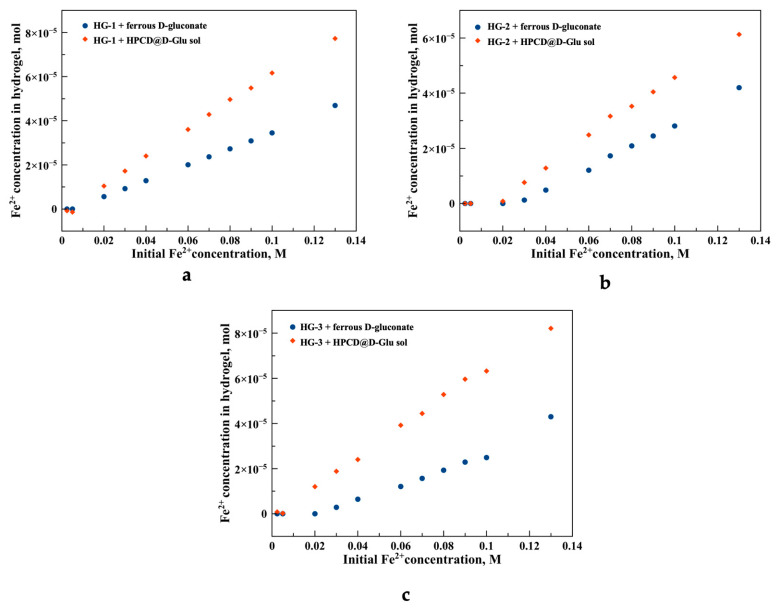
PMSSO + ferrous D-gluconate and PMSSO + ferrous D-gluconate and HPCD complexes’ sorption curves with Fe^2+^ concentration from 0.0024 M to 0.13 M HG-1 (**a**), HG-2 (**b**), and HG-3 (**c**). Ferrous D-gluconate: HPCD ratio was 1:1. pH 4.0 (0.1 mM HCl), 22 °C.

In order to achieve high loading capacity and evaluate the role of hydrogel structure in sorption, we have compared sorption curves for HG-1, HG-2 and HG-3 loaded with ferrous D-Gluconate and HPCD@D-Glu sol. Figure 3 demonstrates sorption curves for initial hydrogels and ferrous D-gluconate (blue dots) and for HPCD@D-Glu sol (red dots). 

The obtained data show an increase in the sorption capacity of hydrogels for HPCD@D-Glu sol in comparison with initial ferrous D-gluconate (Table 3). Thus, loading pharmaceuticals into HPCD increases the sorption capacity of PMSSO hydrogels by approximately twofold. For iron compounds used to treat anemia, it is a favorable phenomenon because it allows for lower dosages and less adverse effects.

**Figure 4:**
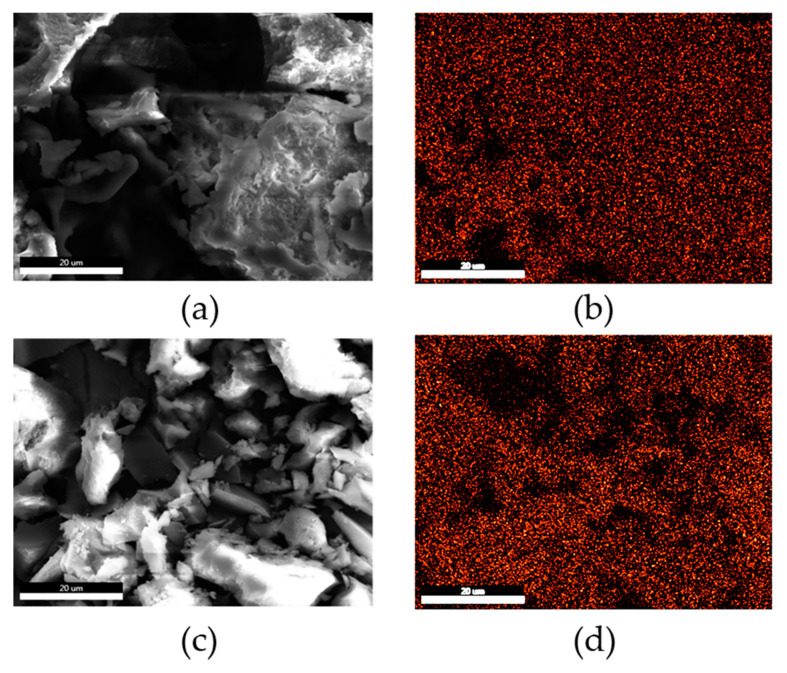
SEM (**a**,**c**) and EDX (**b**,**d**) images of HG-1@HPCD@D-Glu (**a**,**b**) and HG-2@HCPD@G-Glu (**c**,**d**).

Simultaneously, there is a link between sorption capacity and hydrogel structure. Because of the organic nature of HPCD, HG-3 with no additional inorganic linkages should have the strongest affinity for it. In the experiment, we confirmed this hypothesis: when compared to the initial ferrous D-Gluconate, HG-3 has twice the capacity. The capacity of HG-1 and HG-2 containing silicate units increased as well, but not as dramatically. The capacity of HG-2, which contains a greater amount of silicate units, increased less than hydrogel 1. The obtained results are confirmed by PXRD data (Figure S6) and microphotographs and EDX mapping (Figure 4) of HG-1@HPCD@D-Glu and HG-2@HCPD@G-Glu samples. 

According to the EDX data, complex formation with HPCD lead to the more uniform distribution of iron atoms through the PMSSO hydrogel body. Thus, complex formation with HPCD is able to significantly improve the iron atoms distribution in hydrogels and their sorption properties with respect to ferrous D-gluconate. All of this indicates the prospects for further development of this drug delivery system.

### 2.4. Study of Iron Release from the PMSSO-Based Systems

**Figure 5:**
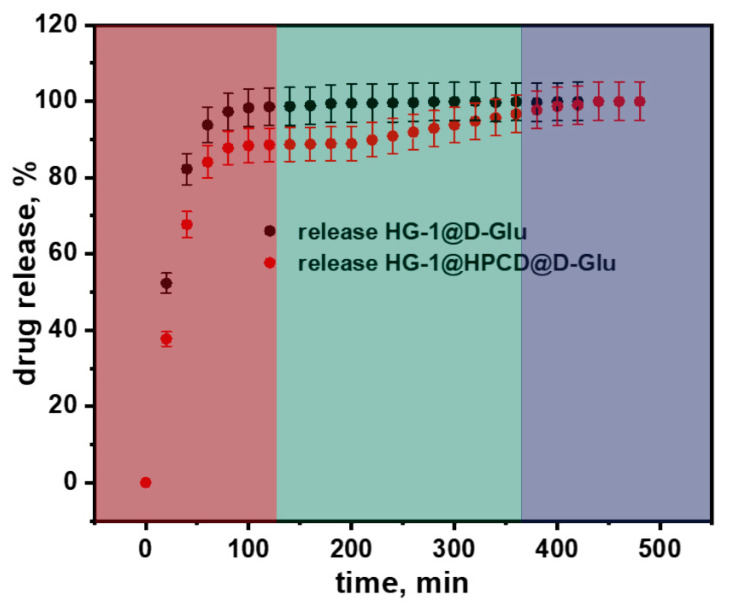
Release curves of iron ions from the delivery systems HG-1@D-Glu (black dots) and HG-1@HCPD@G-Glu (red dots). Conditions: 0.08 M HCl + 0.03 M NaCl, pH = 1.1 (red area), 0.05 M KH_2_PO_4_ + 0.05 M NaOH, pH = 6.8 (green area), 0.07 M KH_2_PO_4_ + 0.07 M Na_2_HPO_4_, pH = 7.4 (blue area).

It is known that the kinetics of release of the active compound from the carrier can play a key role in ensuring optimal pharmacokinetic parameters. In this work, we have studied the kinetics of the release of iron ions from the HG-1@D-Glu and HG-1@HCPD@G-Glu samples. In the experiment, a sequential change of the buffer solution took place in accordance with the digestive media: gastric medium (GM) (0.08 M HCl + 0.03 M NaCl), intestinal medium (CI) (0.05 M KH_2_PO_4_ + 0.05 M NaOH), and deep intestinal medium (DIM) (0.07 M KH_2_PO_4_ + 0.07 M Na_2_HPO_4_). The drug release curves are presented in Figure 5. 

HG-1@D-Glu is characterized by the rapid release of iron ions in an acidic environment (Figure 5, black dots). In contrast, a significant proportion of iron ions is released from the HG-1@HCPD@G-Glu under intestinal conditions (Figure 5, red dots). There are two clearly indicated areas on the curve: the first one could correspond to the release of iron from the hydrogel, while the second one could correspond to the release of iron-loaded HPCD from the hydrogel. In order to describe the behavior of the systems more accurately, the kinetic curves were analyzed within the framework of the most relevant models [22,23,24].

The zero-order model describes the release of a drug from systems for which the rate of release is independent of its concentration. This behavior is typical, for example, for slowly soluble matrices. The first-order model describes the release of drugs from porous matrices. The Higuchi model is based on the diffusion mechanism and is used to describe the release of a drug from an insoluble or partially soluble matrix. The Korsmeyer–Peppas model allows us to determine the type of diffusion during drug release.

An important indicator when determining the kinetic model is the correlation coefficient (R^2^). Table 4 shows the R^2^ values of mathematical processing of the initial section of the release curve from the D-gluconate + hydrogel 1 system.

The processing of the kinetic curve in mathematical models of 0 and 1 order, as well as Higuchi and Korsmeyer–Peppas, enabled us to identify the most likely release mechanism. The curve was found to be most accurately described by the Higuchi model, which corresponds to drug release from semi-solid/solid matrix systems. The literature details the applicability of the model for a variety of drug delivery methods, including silicon carriers [25].

On the other hand, mathematical processing of the release curves from the HG-1@HPCD@D-Glu 1 system was carried out in two sections in order to disclose if there are any differences of mechanism of cargo release. The correlation coefficients are shown in Table 5.

Based on the values obtained, the Higuchi model is the most reliable for characterizing the first part of the curve, which corresponds to the release of the HPCD@D-Glu complex from the HG-1. The second area, which corresponds to the release of iron from HPCD, is consistently described by all mathematical models; therefore, this topic is still up for debate. However, an examination of the literature on the kinetics of the release of the “guest” molecules from cyclodextrin tori revealed that the most plausible from a physico-chemical point of view is the first order [26].

## 3. Conclusions

A proposed oral delivery system for iron compounds should be biocompatible and possess a high drug sorption capacity. In this study, we investigated the possibility of using PMSSO hydrogels with varied amounts of inorganic crosslinking as carriers for iron-containing complexes. Preliminary complex formation with HPCD, followed by loading into hydrogels, results in significantly enhanced sorption capacity as well as the proper distribution of iron ions throughout the gel matrix. 

Compared to previously published results on silicon-based carriers [27] and mucoadhesive microspheres [8], PMSSO hydrogels provide several benefits. According to the Congo red test these gels provide up to 4.5 µmol/g sorption capacity with a specific surface area more than 600 m^2^/g, which makes PMSSO hydrogels an attractive drug delivery system for oral administration. To improve the loading effectiveness of iron compounds into hydrogels, we developed guest–host complexes with HPCD. In separate experiments it was proved that the ferrous D-gluconate complex is formed in a 1:1 ratio and can be effectively loaded to the HG-1, HG-2 and HG-3. 

We investigated the behavior of the obtained systems in a media that simulated the digestive tract. The sequential change of buffer solutions from stomach to intestinal helped highlight the slowing of iron release from the hydrogel when complexes with cyclodextrin were introduced. The release from hydrogels is most likely caused by the Higuchi mechanism, whereas the release of iron from cyclodextrin complexes is most likely caused by first-order kinetics. Completed experiments suggest that such systems could be used to distribute iron-containing drugs.

## 4. Materials and Methods

### 4.1. Materials

We used the following reagents: methyltriethoxysilane (Reakhim, Moscow, Russia), hydrochloric acid (HCl, 35%, 36.46 g/mol, 1.18 g/cm^3^, purity ≥ 99%, Khimmed, Moscow, Russia), anhydrous sodium hydroxide (NaOH, 39.99 g/mol, purity ≥ 95%, Komponent Reaktiv, Moscow, Russia), 2-hydroxypropyl-β-cyclodextrin with degree of substitution 6.0–8.0 ((C_6_H_9_O_5_)_7_(C_3_H_7_O)n, 1134.98 + n*58 g/mol, Zibo Qianhui Biological Technology Co., Zibo, China), Iron (III) chloride hexahydrate (FeCl_3_ × 6H_2_O), 270.30 g/mol, purity 97%, Sigma-Aldrich, Saint Louis, MO, USA), ferrous gluconate dihydrate (Fe[HOCH_2_(CHOH)_4_CO_2_]_2_ × 2H_2_O, 99.7%, Ruschim, Moscow, Russia), sodium silicate sol solution (purity > 95%, Spektrkhim, Moscow, Russia), sodium chloride (NaCl, 58.44 g/mol, purity 99.9%, Reakhim, Moscow, Russia), Potassium phosphate monobasic (KH_2_PO_4_, 136.09 g/mol, purity ≥ 99%, Sigma Aldrich, St. Louis, MO, USA), and Sodium phosphate dibasic (Na_2_HPO_4_, 141.96 g/mol, purity ≥ 99%, Sigma Aldrich, St. Louis, USA).

### 4.2. Methods

#### 4.2.1. Synthesis of Polymethylsilsesquioxane (PMSSO) Hydrogels

Synthesis of polymethylsilsesquioxane sol. A solution of sodium hydroxide (25.4 g, 0.634 mol) in water (300 mL) was added to a stirred mixture of methyltriethoxysilane (112.9 g, 0.634 mol). The reaction mixture was stirred for 30 min to give a colorless solution of sol. The synthesis was carried out at room temperature.

Synthesis of polymethylsilsesquioxane hydrogel 1. A sodium silicate sol (70.3 g, 0.575 mol) in water (68 mL) was added to a stirred polymethylsilsesquioxane sol (139 g). A solution of acetic acid (25.2 g, 0.421 mol) in water (140 mL) was added rapidly under stirring. The gel obtained was left for 20 h for aging and then, was rinsed on a filter with a small amount of a solution of 36.5% hydrochloric acid to a neutral reaction (pH = 7). The synthesis was carried out at room temperature.

Synthesis of polymethylsilsesquioxane hydrogel 2. A solution of sodium silicate sol (70.3 g, 0.575 mol) in water (68 mL) was added to a stirred polymethylsilsesquioxane sol (69.2 g). A solution of acetic acid (13.2 g, 0.221 mol) in water (113 mL) was added rapidly under stirring. The gel obtained was left for 20 h for aging and then was rinsed on a filter with a small amount of a solution of 36.5% hydrochloric acid to a neutral reaction (pH = 7). The synthesis was carried out at room temperature.

Synthesis of polymethylsilsesquioxane hydrogel 3. The synthesis was carried out in accordance with the methodology detailed in [14]. A solution of acetic acid (24.1 g, 0.389 mol) in water (218 mL) was added rapidly to polymethylsilsesquioxane sol (300 g) under stirring. The gel obtained was left for 20 h for aging and then was rinsed on a filter to a neutral reaction (pH = 7). The synthesis was carried out at room temperature.

**Scheme 1:**
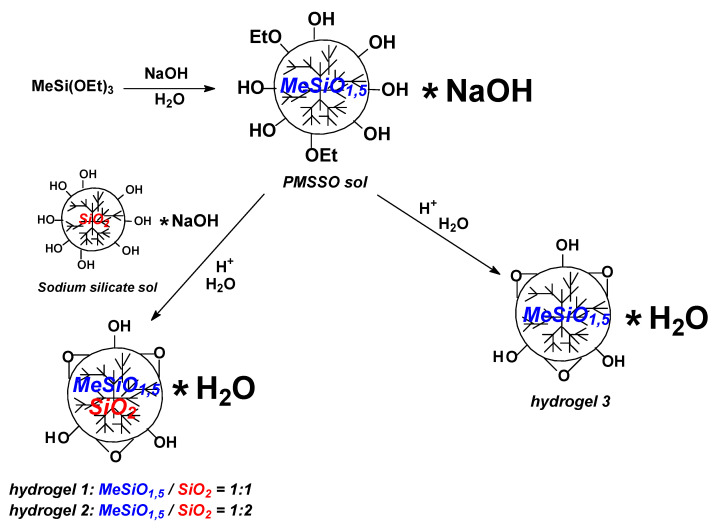
Synthesis of PMSSO hydrogels.

The full synthesis is presented on Scheme 1.

#### 4.2.2. Nuclear Magnetic Resonance

The ^29^Si NMR spectra of PMSSO sol were registered on a Bruker Avance III 400 spectrometer (Bruker, Elltingen, Germany). The analysis of the PMSSO sol was carried out in d-ethanol upon addition of Cr(acac)_3_. The analysis of PMSSO gels was carried out using a solid–state sensor with rotation at a magic angle with a frequency of 8 kHz using cross-polarization and decoupling from 1 H.

The UV-spectra were recorded with an Ultrospec 2100 pro instrument (Amersham Biosciences, Amersham, UK) within a wavelength range of 200–500 nm in a 1 mL quartz cell (Hellma Analytics, Müllheim, Germany). 

The IR-spectra were recorded using a Tensor 27 Fourier-transform IR spectrometer (Bruker, Elltingen, Germany) equipped with an MCT detector cooled with liquid nitrogen and a thermostat (Huber, Offenburg, Germany). The measurements were carried out in a thermostated BioATR II cell with an attenuated total internal reflection (ATR, Bruker, Germany) using a single-reflection ZnSe crystal at 22 °C and a constant purge rate of the system with dry air using a Jun-Air apparatus (Jun-Air, Redditch, UK). An aliquot portion of the sample (40 μL) was applied on the crystal of the ATR cell; the spectrum was recorded thrice in the range from 4000 to 950 cm^−1^ with a resolution of 1 cm^−1^; it was scanned 70 times and averaged. The background signal was recorded similarly. The Opus 7.0 software was used to analyze the spectra.

Scanning electron microscopy (SEM) and energy-dispersive X-ray spectrometry (EDXs) were performed using a FEI Helios G4 CX dual beam scanning electron microscope (Hillsboro, OR, USA) equipped with system EDAX Octane Elite Super with SPI and TEAM 3D IQ 3.11 software. Preliminarily, a thin layer of Ag (10 nm) was coated to samples using SPI Supplies (West Chester, PA, USA). The elements mapping was collected for Fe and Si from 150 μm^2^ using the SEM-EDX mode with TEAM attachments.

Powder X-ray diffraction (PXRD) patterns were collected on a Thermo ARL X’TRA powder diffractometer (CuKα radiation, λ = 1.5418 Å, Bragge-Brentano geometry, Peltier-cooled CCD detector) at TR. PXRD data were collected at room temperature over the 10–50° 2Θ range with 0.02° steps.

Freeze-drying. Samples were freeze-dried for two days at −60 °C (Edwards 5, BOC Edwards, Atlas Copco Group, Stockholm, Sweden).

Calibration curves. The initial sample of FeCl_3_ × 6H_2_O with concentration 0.27 M was diluted with a buffer solution (0.0001 M HCl pH 4.0) to FeCl_3_ × 6H_2_O concentration from 0 to 0.0001 M. UV spectra of the obtained solutions were taken. Then, the degree of binding of FeCl_3_ × 6H_2_O to the carrier was calculated by Equation (1). The experiment was triplicated with R^2^ = 0.9899.

(1) $$N=\frac{D_{init}-D_{fin}}{D_{init}}\times 100\%$$

where *N* is the degree of binding of FeCl_3_ × 6H_2_O to hydrogel and *D_init_* and *D_fin_* are the values of the initial and final absorbance.

The initial sample of ferrous D-gluconate with concentration 0.24 M was diluted with a buffer solution (0.0001 M HCl pH 4.0) to a concentration from 0 to 0.24 M. IR spectra of the obtained solutions were taken. Then, the degree of binding of ferrous D-gluconate to the carrier was calculated by Equation (2). The experiment was triplicated with R^2^ = 0.9538.

(2) $$N=\frac{I_{init}-I_{fin}}{I_{init}}\times 100\%$$

where *N* is the degree of binding of ferrous D-gluconate to hydrogel and *I_init_* and *I_fin_* are the values of the initial and final 1539 cm^−^^1^ intensity.

Preparation of FeCl_3_ × 6H_2_O + HPCD complexes (HPCD@FeCl_3_ kn) by the kneading method. FeCl_3_ × 6H_2_O and HPCD in 1:5 quantities were treated with a small amount of ethanol: water mixture. The slurry was kneaded for 10 min and then freeze-dried.

Preparation of ferrous D-gluconate + HPCD (HPCD@D-Glu sol). The HPCD solution (0.0001 M HCl pH 4.0) was added to ferrous D-gluconate solution (0.0001 M HCl pH 4.0). Concentration of ferrous D-gluconate was 0.04 M, HPCD concentration was varied from 0 to 0.08 M. Complexes were incubated for 1 h, 37 °C, 150 rpm. 

Determination of the adsorption capacity of the hydrogels for HPCD@D-Glu. Complexes HPCD@D-Glu sol were prepared diluting to concentrations from 0.0024 to 0.13 M with a buffer solution (0.0001 M HCl pH 4.0) from the initial complex solution with ferrous D-gluconate and HPCD concentrations 0.13 M. Complexes were incubated for 1 h, 37 °C, 150 rpm. Then 1 mL of complexes were added to 100 mg of hydrogels. Mixture was incubated for 1 h, 37 °C, 150 rpm, then centrifuged for 20 min, 6000 rpm. 

**Scheme 2:**
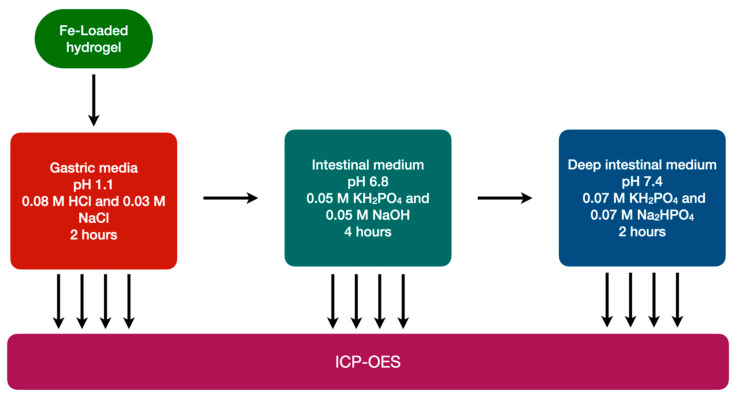
The pipeline of the Fe-release experiment in the gastro-internal tract media in vitro.

Fe release experiments. The release experiment was carried out with simulation in three digestive media in order to imitate the gastro-internal tract in vitro. The main pipeline of the experiment is presented on Scheme 2. 

Samples of the loaded hydrogel (20 mg) were added to 10 mL of the gastric media pH 1.1, containing 0.08 M HCl and 0.03 M NaCl. After 2 h of incubation at 37 °C, the samples were moved to the intestinal medium (IM) that consisted of 0.05 M KH_2_PO_4_ and 0.05 M NaOH (pH 6.8) for 4 h followed by the deep intestinal medium (DIM) 0.07 M KH_2_PO_4_ + 0.07 M Na_2_HPO_4_ (pH 7.4) for 2 h. 

Every 20 min, a sample of 2 mL was taken, after which the sample was filled with a fresh portion of the medium.

#### 4.2.3. Statistical Analysis

All experiments were triplicated, and the results were expressed as the mean value ± standard deviation, SD (n = 3). AtteStat 3.04 for Microsoft Excel was used for statistical analysis. Significance was analyzed by the Mann–Whitney test, with *p* ≤ 0.05 considered statistically significant.

## Figures and Tables

**Table 1:** Sample names and compositions.

Sample Name	Composition
HG-1	1:1 MeSiO_1.5_/SiO_2_ links molar ratio
HG-2	1:2 MeSiO_1.5_/SiO_2_ links molar ratio
HG-3	No extra SiO_2_ links
HPCD@D-Glu sol	Guest–host complex of ferrous D-Gluconate and HPCD in molar ratio 1:1 obtained from the aqueous solution
HPCD@FeCl_3_ sol	Guest–host complex of FeCl_3_ and HPCD in molar ratio 1:5 obtained from the aqueous solution
HPCD@FeCl_3_ kn	Guest–host complex of FeCl_3_ and HPCD in molar ratio 1:5 obtained from the kneading
HG-1@D-Glu	Hydrogel HG-1 loaded with ferrous D-Gluconate
HG-1@HPCD@D-Glu	Hydrogel HG-1 loaded with complex HPCD@D-Glu sol

**Table 2:** PMSSO hydrogels sorption capacity and specific surface area.

Sample Name	Composition	Sorption Capacity, µmol/g	Specific Surface Area, m^2^/g
HG-1	1:1 MeSiO_1.5_/SiO_2_ links molar ratio	0.9	278.7 ± 4.5
HG-2	1:2 MeSiO_1.5_/SiO_2_ links molar ratio	0.2	313.2 ± 5.6
HG-3	No extra SiO_2_ links	4.5	620 ± 10.5

**Table 3:** The 0.13 M ferrous D-gluconate and HPCD@FeCl_3_ sol complexes with 0.13 M ferrous D-gluconate sorption by PMSSO hydrogels, pH 4.0 (0.1 mM HCl), 22 °C.

Hydrogel	Sorption Capacity for Ferrous D-Gluconate, %	Sorption Capacity for HPCD@G-Glu Sol, %
HG-1	37 ± 4	59 ± 6
HG-2	35 ± 4	47 ± 5
HG-3	33 ± 3	63 ± 6

**Table 4:** R^2^, obtained by mathematical processing of the initial section of the release curve from the system, within the framework of several mathematical models of ferrous D-gluconate release from the hydrogel 1.

Model	R^2^
Zero-order	0.85
First-order	0.8119
Higuchi model	0.9806
Korsmeyer–Peppas model	0.9389

**Table 5:** R^2^, obtained by mathematical processing of the initial section of the release curve from the system, within the framework of several mathematical models of HPCD@D-Glu release from the HG-1@HPCD@D-Glu 1.

Model	R^2^, Hydrogel Release Area	R^2^, HPCD Release Area
Zero-order	0.9115	0.9981
First-order	0.834	0.9968
Higuchi model	0.9803	0.9984
Korsmeyer–Peppas model	0.9522	0.9962

## Data Availability

Data are available upon request.

## Supplementary Materials

The following supporting information can be downloaded at: https://www.mdpi.com/article/10.3390/gels10090564/s1, Figure S1: Structure and ^29^Si NMR spectrum of PMSSO sol; Figure S2: Structure and ^29^Si NMR spectra of HG-1 (**a**), HG-2 (**b**) and HG-3 (**c**).; Figure S3: FTIR spectra of hydrogels in water; Figure S4: PRXD data for the (**a**) HPCD@ FeCl_3_ sol freeze-dried and (**b**) HPCD@ FeCl_3_ kn. Molar ration guest–host is 1:5.; Figure S5: PRXD data for HPCD@D-Glu sol complex. Guest–host molar ratio 1:1; Figure S6: PXRD data for HG-1@HPCD@D-Glu (**a**) and HG-2@HPCD@D-Glu (**b**).
