# Supplementary material for: Cyclodextrin—Polymethylsilsesquioxane Combined System as a Perspective Iron Delivery System for Oral Administration
Source: Gels. 2024 Aug 30;10(9):564. doi: 10.3390/gels10090564 (PMC11431624; doi:10.3390/gels10090564)
Supplement: Supplementary file 1 [file gels-10-00564-s001.zip › gels-3163221-supplementary.pdf]

Supplementary materials

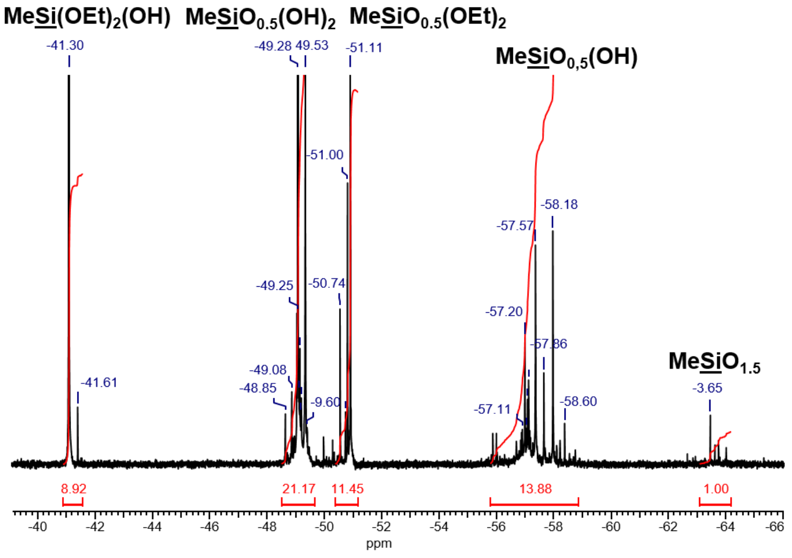

Figure S1. Structure and ^{29}Si NMR spectrum of PMSSO sol.

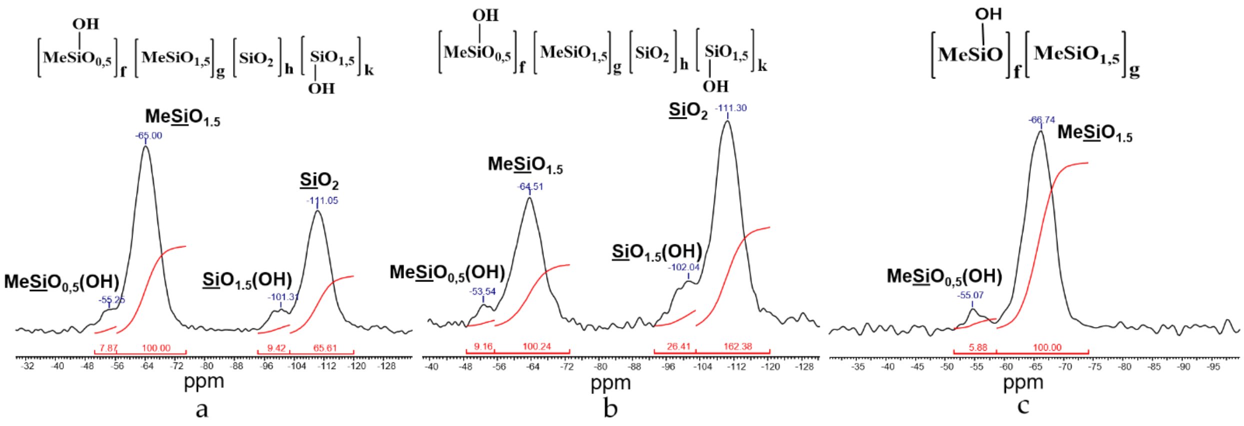

Figure S2. Structure and ^{29}Si NMR spectra of HG-1 **(a)**, HG-2 **(b)** and HG-3 **(c)**.

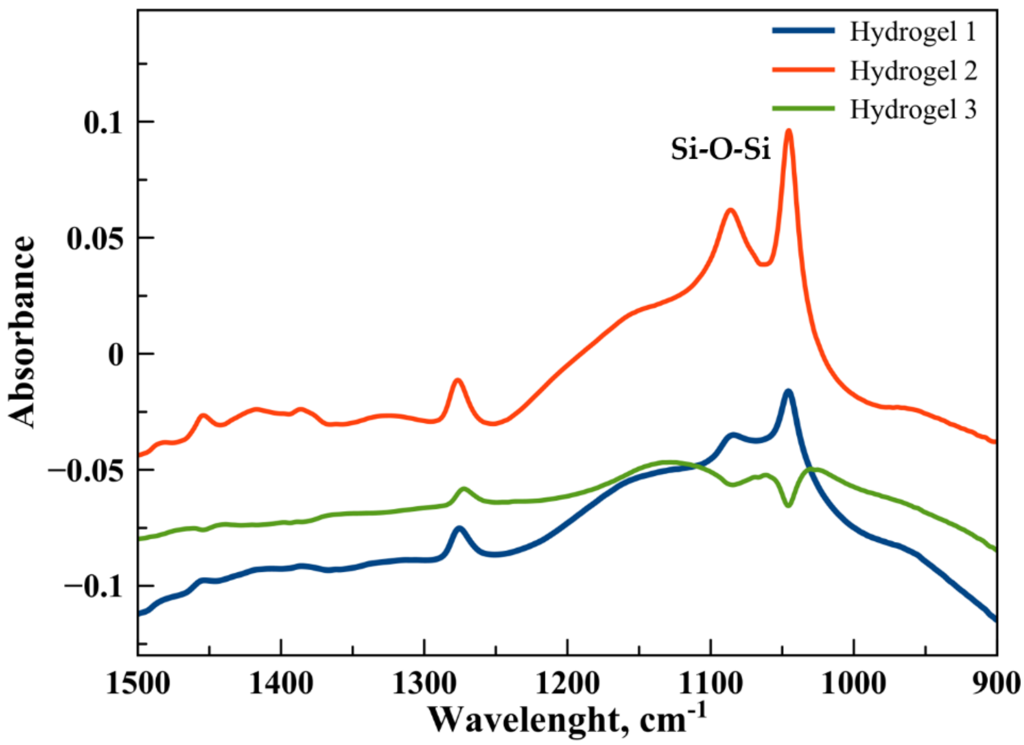

Figure S3 FTIR-spectra of hydrogels in water, 22 °C.

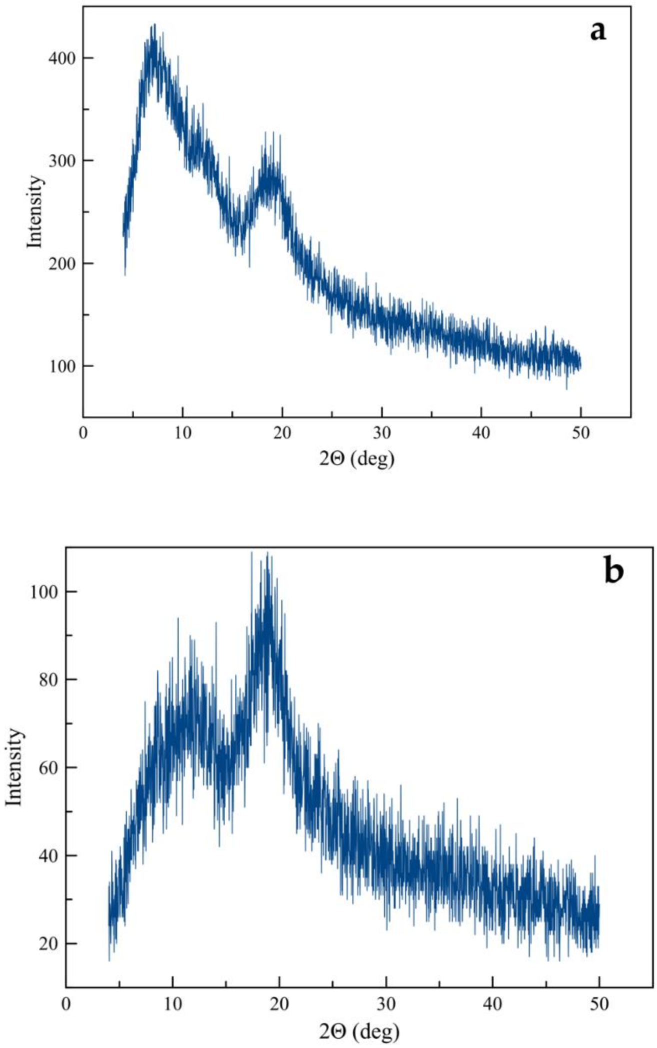

Figure S4. PRXD data for the **(a)** HPCD@ $FeCl_3$ sol freeze-dried and **(b)** HPCD@ $FeCl_3$ kn. Molar ration guest-host is 1:5.

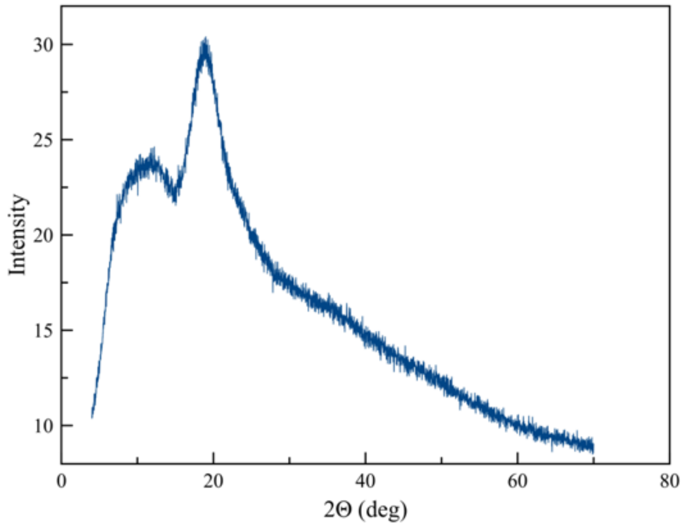

Figure S5. PRXD data for HPCD@D-Glu sol complex. Guest-host molar ratio 1:1.

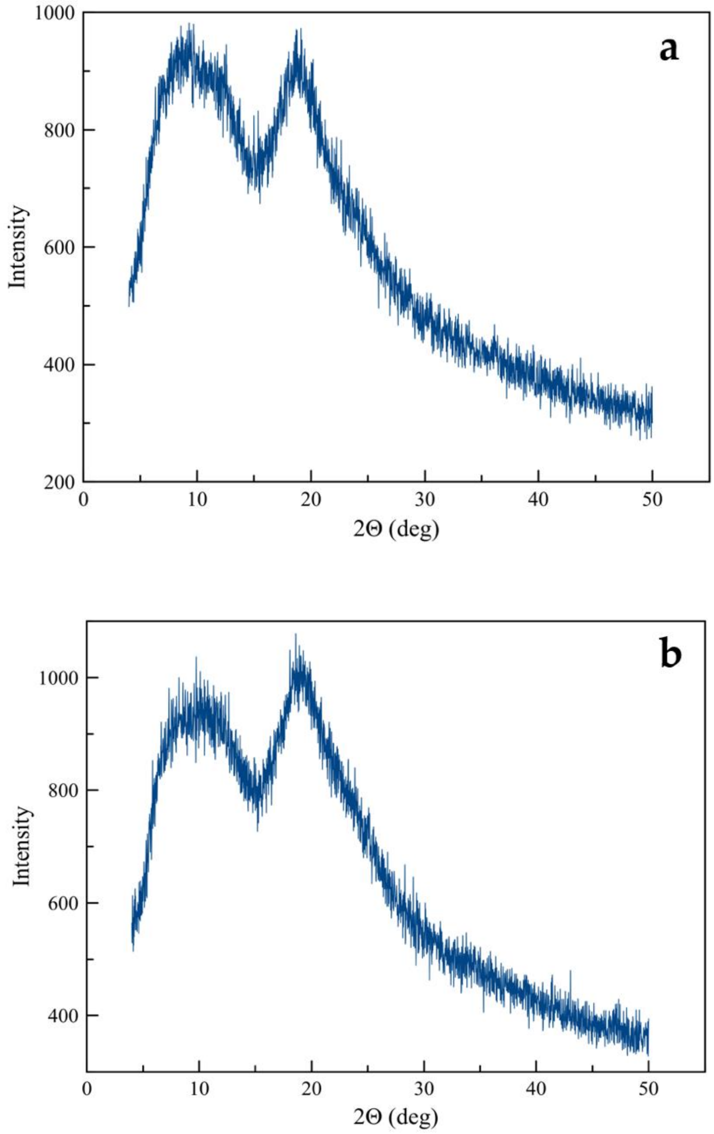

Figure S6. PXRD data for HG-1@HPCD@D-Glu **(a)** and HG-2@HPCD@D-Glu **(b).**

Atomic emission spectrometry methodic

The total iron content in all samples was determined using the inductively coupled plasma atomic emission spectrometry (ICP-OES) on an Agilent 720 instrument (Agilent, Mulgrave, Australia). Calibration standards ranging from 20 to 10,000 ppb were prepared using a certified reference material, iron 10,000 ppm standard from Inorganic Ventures (Christiansburg, VA, USA), with 0.3 M nitric acid as the medium. The data obtained from the spectral lines at 238.204, 239.562, and 259.940 nm were averaged. The relative standard deviation (in RSD,%) did not exceed 3%. Prior to analysis, all samples were ten-fold diluted to reduce the influence of salt matrices. The stability of the plasma during analysis was assessed by measuring the intensity ratio of the magnesium ionic and atomic lines, Mg II 280.270 nm and Mg I 285.213 nm, respectively, using a 10 ppm solution which mixed with scandium 20 ppm as an internal standard. Two parallel samples were analyzed. The accuracy of the analysis was evaluated through a spiked sample analysis. All necessary data were calculated following the nomenclature outlined in the IUPAC Recommendations 1994 for presenting chemical analysis results.

Kinetic curves were described with kinetic models of zero and first order, Higuchi and Korsmeyer-Peppas models. The most suitable model for each system was determined by the highest value of the correlation coefficient (R^2). In case of release curve of hydrogel 1 + ferrous D-gluconate system the dots corresponding to the first 0-80 min were analyzed.

Two clearly indicated areas on the hydrogel 1 + ferrous D-gluconate + HPCD release curve were analyzed. The first area (release of iron from the hydrogel) contains dots, corresponding to the first 0-80 min, while the second one to (release of iron-loaded HPCD from the hydrogel) contains dots, corresponding to the first 200-440 min.
